# Prognostic Value of Gene Signatures and Proliferation in Lymph-Node-Negative Breast Cancer

**DOI:** 10.1371/journal.pone.0090642

**Published:** 2014-03-05

**Authors:** Kristin Jonsdottir, Jörg Assmus, Aida Slewa, Einar Gudlaugsson, Ivar Skaland, Jan P. A. Baak, Emiel A. M. Janssen

**Affiliations:** 1 Department of Pathology, Stavanger University Hospital, Stavanger, Norway; 2 Centre for Clinical Research, Haukeland University Hospital, Bergen, Norway; 3 Free University, Amsterdam, The Netherlands; University of Turin, Italy

## Abstract

**Introduction:**

The overall survival rate is good for lymph-node-negative breast cancer patients, but they still suffer from serious over- and some undertreatments. Prognostic and predictive gene signatures for node-negative breast cancer have a high number of genes related to proliferation. The prognostic value of gene sets from commercial gene-expression assays were compared with proliferation markers.

**Methods:**

Illumina WG6 mRNA microarray analysis was used to examine 94 fresh-frozen tumour samples from node-negative breast cancer patients. The patients were divided into low- and high-risk groups for distant metastasis based on the MammaPrint-related genes, and into low-, intermediate- and high-risk groups based on the recurrence score algorithm with genes included in Oncotype DX. These data were then compared to proliferation status, as measured by the mitotic activity index, the expressions of phosphohistone H3 (PPH3), and Ki67.

**Results:**

Kaplan-Meier survival analysis for distant-metastasis-free survival revealed that patients with weak and strong PPH3 expressions had 14-year survival rates of 87% (*n* = 45), and 65% (*n* = 49, *p* = 0.014), respectively. Analysis of the MammaPrint classification resulted in 14-year survival rates of 80% (*n* = 45) and 71% (*n* = 49, *p* = 0.287) for patients with low and high risks of recurrence, respectively. The Oncotype DX categorization yielded 14-year survival rates of 83% (*n* = 18), 79% (*n* = 42) and 68% (*n* = 34) for those in the low-, intermediate- and high-risk groups, respectively (*p* = 0.52). Supervised hierarchical cluster analysis for distant-metastasis-free survival in the subgroup of patients with strong PPH3 expression revealed that the genes involved in Notch signalling and cell adhesion were expressed at higher levels in those patients with distant metastasis.

**Conclusion:**

This pilot study indicates that proliferation has greater prognostic value than the expressions of either MammaPrint- or Oncotype-DX-related genes. Furthermore, in the subgroup of patients with high proliferation, Notch signalling pathway genes appear to be expressed at higher levels in patients who develop distant metastasis.

## Introduction

It is essential that the decision of whether or not to treat breast cancer patients is made as accurately as possible. It is therefore of utmost importance to be able to properly distinguish between those breast cancer patients who would benefit from adjuvant systemic therapy and those who could be spared such treatment. Different guidelines [1]–[3] have been developed to assist clinicians in making treatment decisions. These guidelines are often based on a combination of both clinical and pathological parameters that provide information about both the prognosis and prediction for therapy response. Unfortunately, even with these guidelines, over- and undertreatment of lymph node (LN)-negative breast cancer patients still occurs. For example, the St. Gallen guidelines advise adjuvant treatment in 85% of all node-negative breast cancers, even though only 15–20% would die without treatment. Therefore, new parameters are needed to complement or replace the current clinicopathological features to ensure better characterization and treatment of breast tumours.

Microarray analysis can provide an expression profile of all genes in a tumour, thereby giving an impression of all of the active and inactive processes in that tumour. Studies on the gene-expression signatures of breast tumours have led to a new classification of breast cancers into at least five different subtypes with very different prognoses [4]. Supervised transcript profiling analyses have subsequently been used to develop standardized molecular prognostic indicators, such as MammaPrint and Oncotype DX [5], [6]. These commercially available tests combine data from different biological pathways to provide information about both the prognosis and response to endocrine therapy and chemotherapy. Oncotype DX low- and intermediate-risk patients have shown significant benefit from tamoxifen treatment, while patients with a high risk of distant breast cancer recurrence gain additional benefit from chemotherapy [7]. Interestingly, the genes that contributed most to these results were five proliferation genes (those encoding cyclin B1, Ki67, Myb-related protein B, survivin and serine/threonine-protein kinase 6), and those encoding the progesterone and **o**estrogen receptors (PR and ER, respectively) [6].

MammaPrint is a 70-gene expression assay that distinguishes between patients with high and low risks of distant recurrence using genes associated with proliferation, metastases, stromal invasion and angiogenesis. The first study was found to distinguish LN-negative breast cancer patients aged <55 years. The validations of this signature have included patients of all ages with both LN-positive and -negative disease, and it has been shown to predict relapse better than traditional histopathological features [8], [9]. Interestingly, the gene signatures correlated with outcome contain high numbers of genes related to proliferation. A recent study found that a signature based upon cell-cycle-related genes alone was a more accurate predictor of breast cancer clinical outcome than another FDA-approved signature containing many more genes [10]. Another study [11] found that the simplest model for defining the risk score was the expression of a single proliferation gene, which yielded similar or an even better performance than models fitted from genome-wide data, and outperformed classical factors such as histological grade. These results are in agreement with previous retrospective and prospective studies involving large numbers of LN-negative breast cancer patients showing that proliferation measured by a thymidine labelling index, mitotic activity index (MAI), or phosphohistone H3 (PPH3) labelling are stronger prognosticators than classical predictors (reviewed in [12]). Moreover, two independent studies found that adjuvant chemotherapy was significantly beneficial for patients with rapidly proliferating tumours, but not for those with slowly proliferating tumours [13], [14]. High rates of cell proliferation are correlated with shorter cancer survival [15], [16]. Hence, it is of great interest to compare the prognostic value of the classical pathological prognosticators, proliferation markers, genes included in MammaPrint and Oncotype DX assays, and other new potential biomarkers for breast cancer.

The aim of this pilot study was, to determine whether gene expression can add prognostic information for subgroups of patients with tumours with low or high proliferative activity. Since proliferation measured by using MAI or PPH3 labelling has repeatedly proven to be the best prognosticator in LN-negative breast cancer (high sensitivity with little overtreatment), the rationale to commence with proliferation assessment for treatment decision-making may be logical.

## Material and Methods

### Patients

This study was approved by the Regional West committee for medical and health research ethics, the Norwegian Science Data Service and the Norwegian Data Inspectorate. None of the patients were required to provide written informed consent to participate since they had been diagnosed in the period 1993–1997. The regional ethics committee has approved this study. All insights in a patient's journal were monitored electronically, and all except the treating physician were required to state the reason why they needed to read that patient's journal. This log was always open for the patient to view. All patients were treated according to the national guidelines of the Norwegian Breast Cancer Group at the time of diagnosis. Fresh-frozen tumour tissue for hormone receptor determination was collected from 235 individual patients, of which 135 patients were LN negative. Some of these patients had to be excluded because of bilateral disease (*n* = 3), previous breast tumours (*n* = 8), lack of follow-up (*n* = 3), lack of adequate material (*n* = 10) and poor-quality RNA (*n* = 17). This left 94 patients with adequate material and follow-up. Of these patients, 13 received endocrine therapy and 10 received chemotherapy. Of the 77 oestrogen receptor alpha (ERα)-positive patients, 12 received endocrine therapy and 4 received chemotherapy.

### Histopathology and Immunohistochemistry

The main tumour tissue was fixed in buffered 4% formaldehyde and then embedded in paraffin. Sections were cut at a thickness of 4 µm and stained with haematoxylin, erythrosin and saffran. The histological type was assessed according to World Health Organization criteria [17], and the tumour grade was assessed according to the Nottingham modification [18]. The MAI and PPH3 labelling were assessed as described previously [19], [20].

Immunohistochemistry (IHC) was used to detect ERα, progesterone receptor (PR), human epidermal growth factor receptor 2 (HER2), PPH3, Ki67 and cytokeratin 5/6 (CK5/6). The methods were based on DAKO technology as described previously [21]. ERα was scored positive if ≥1% of tumour cells exhibited nuclear staining, while all others were scored negative. PR was scored as positive when nuclear staining was present in ≥10% and scored negative when <10% of the tumour cells had nuclear staining. The DAKO Hercep-Test scoring protocol was used for measuring HER2, with cases scored as 3+ considered to be positive. The cases that were scored as 2+ were further validated with fluorescence in situ hybridization using the Pathvysion HER2 DNA probe kit (Abbott Laboratories, Abbott Park, IL, USA) for HER2-neu amplification. The manufacturer's protocol was followed. Triple-negative breast cancers (TNPs) were defined as being negative for ERα (0%), PR (<10%) and HER2 (− and +). The semiautomatic interactive computerized QPRODIT system (Leica, Cambridge, UK) was used for measuring the percentage of Ki67-positive cells, as described by Gudlaugsson et al. [22]. CK5/6-positive tumour cells were scored using a continuous scale of 0–100%, where in the final analysis all tumours with any CK5/6 staining in tumour cells were grouped as being positive, as described previously [23]. All sections were independently scored by two pathologists.

### RNA Isolation/Labelling/Hybridization

All cryosections used for RNA isolations were evaluated by an experienced breast pathologist (E.G.). An area comprising at least 50% tumour cells was isolated by means of macrodissection. At least two 10-µm cryosections were used for total RNA isolation using the MirVANA total RNA isolation kit (Ambion/Applied Biosystems, Austin, TX, USA), according to the protocol provided by the manufacturers. For quality control, all samples were analysed using both the Agilent 2100 Bioanalyzer system (total RNA and small RNA chips) and the NanoDrop spectrophotometer (Thermo Scientific, Wilmington, DE, USA). The microarray experiment was performed using the Illumina iScan device, which uses fluorescence detection of biotin-labelled cRNA. For each sample, 250 ng of total RNA was reverse transcribed, amplified and labelled with biotin-UTP using an Illumina TotalPrep-96 RNA Amplification Kit (version 4393543, Ambion/Applied Biosystems). The quantity of labelled cRNA was measured using the NanoDrop spectrophotometer (Thermo Scientific), whereas the quality and size distribution of the labelled cRNA was assessed using the 2100 Bioanalyzer (Agilent). Finally, 1.5 µg of biotin-labelled cRNA was hybridized to Illumina HumanWG-6 v3 Expression BeadChips according to the manufacturer's protocol.

### Survival Endpoints

Distant-metastasis-free survival (DMFS) was used as the main endpoint for both Kaplan-Meier survival plots and as an event for both univariate and multivariate Cox analysis. Patients were surveyed until the date of last follow-up visit for death from causes other than breast cancer, local or regional recurrences, and the development of a second primary cancer, including contralateral breast cancer. If a patient's status during follow-up indicated a confirmed metastasis without a recurrence date, the last follow-up visit date was used. Age, time to first distant recurrence and survival time were calculated relative to the primary diagnosis date.

### Statistical Analyses

Statistical analyses were conducted using SPSS (version 20.0, SPSS, Chicago, IL, USA) and MATLAB 7.10 (MathWorks, Natick, MA, USA). Differences between patient groups were tested using the log-rank test and Kaplan-Meier survival curves. Cox proportional-hazard analysis (forward, Wald) and hazard ratio (HR) with 95% confidence intervals (CIs) were used to determine the relative importance of the prognostic variables.

The MammaPrint classification and recurrence score (RS) algorithm from Oncotype DX were computed as described by Paik et al. [6], [8] and van de Vijver et al. [6], [8]. The average gene-expression profile of patients (AGPP) with and without distant metastasis was calculated using MammaPrint-related genes. Each sample was then correlated with each of these AGPPs and classified. Receiver operating characteristic (ROC) curve analysis was used with respect to DMFS to determine cut-offs for both indices. The optimal cut-offs were defined as those with an ROC analysis value closest to 1. For Oncotype DX, a second cut-off was defined as the optimal cut-off in the highly sensitive part of the curve, thus defining three risk categories: low, medium and high. In addition, a cluster analysis was performed for the tumours using both Pearson and Spearman correlations, and the two main clusters were defined as high- and low-risk groups. Kaplan-Meier survival analysis was used with the log-rank test to test whether there was a significant difference in DMFS between the different patient groups determined univariately by different risk factors (Table 1). A multivariate analysis was performed including all significant univariate factors from Table 1, according to a Cox regression. This data set is available publicly in the Gene Expression Omnibus: accession number GSE46563.

**Table 1:** Distant-metastasis-free survival in lymph node negative breast cancer patient with Kaplan-Meier survival- and univariate analysis.

Characteristic	Distant metastases			
	Event/at risk (%)	Log-rank P-value	HR	95% CI
**Age**				
<55 years	6/35 (83)	0.099	2.2	0.8–5.5
≥55 years	17/59 (71)			
**Tumour diameter**				
<2 cm	12/56 (79)	0.334	1.9	0.8–4.3
≥2 cm	11/38 (71)			
**Nottingham grade**				
1	3/14 (79)	0.759	1.2	0.4–4.1
2 and 3	20/80 (75)			
**ER**				
Positive ≥1%	18/77 (77)	0.585	1.3	0.5–3.6
Negative	5/17 (71)			
**PR**				
Positive ≥10%	17/78 (78)	0.268	1.4	0.6–3.2
Negative 0–9%	6/16 (63)			
**HER2**				
0–1+	21/82 (74)	0.533	1.6	0.4–6.7
2+–3+	4/14 (83)			
**MAI**				
<10	11/60 (82)	0.041	2.3	1.0–5.2
≥10	12/34 (65)			
**MAI**				
MAI 0–2	2/34 (94)	0.003	6.7	1.5–31.8
MAI 3–9	9/26 (65)		7.7	1.7–34.5
MAI ≥10	12/34 (65)			
**Ki67**				
0–9%	5/39 (87)	0.05	2.6	1.0–7.0
10–100%	18/53 (66)			
**PPH3**				
<13	6/45 (87)	0.014	3.1	1.2–7.8
≥13	17/49 (65)			
**CK5/6**				
Negative	19/82 (77)	0.547	1.4	0.5–6.0
Positive	4/12 (67)			
**Triple Negative**				
Positive	21/89 (76)	0.352	1.4	0.5–4.2
Negative	2/5 (60)			
**Oncotype DX genes**				
Low risk	3/18 (83)	0.522	1.2	0.3–4.4
Medium risk	9/42 (79)		1.8	0.5–6.5
High risk	11/34 (68)			
**MammaPrint genes**				
Good prognosis	9/45 (80)	0.287	1.6	0.7–3.6
Bad prognosis	14/49 (71)			

The free downloadable software package dChip (version 31 March 2009) was used to identify genes related to different clinical features and to the presence or not of distant metastases (survival time was not taken into account). Absolute correlations (including genes with opposing gene profiles) were calculated using ANOVA, with the cut-off for statistical significance set at *p*≤0.01. Gene lists created in this way were used to classify samples by cluster analysis. The classification accuracy was tested by performing a cross-validation analysis (by leave one sample out and reselect genes). The created gene lists were further analysed for Gene Ontology terms using the free software programs Gorilla [24], [25] and DAVID [26], [27]. The same exercise was also performed for the patients with high proliferation only.

## Results

Ninety-four patients with LN-negative breast cancer were included; their clinical features are listed in Table 1. The median age of this group was 60 years, and the median follow-up period was 127 months (range 14–171 months). Twenty-three patients (24%) developed distant metastasis or died from breast-cancer-related disease. The genes involved in the Oncotype DX [6] and the MammaPrint assay [28], and their concordant Illumina names, are presented in Tables S1 and S2 in File S1. Of the 70 genes present in the MammaPrint assay, 62 were identified in the Illumina array, as were all of the genes included in the Oncotype DX assay. Table 1 presents the DMFS and HRs for all of the tumour characteristics; MAI, and PPH3 and Ki67 status appear to be strong prognostic factors in this cohort of samples.

**Figure 1:**
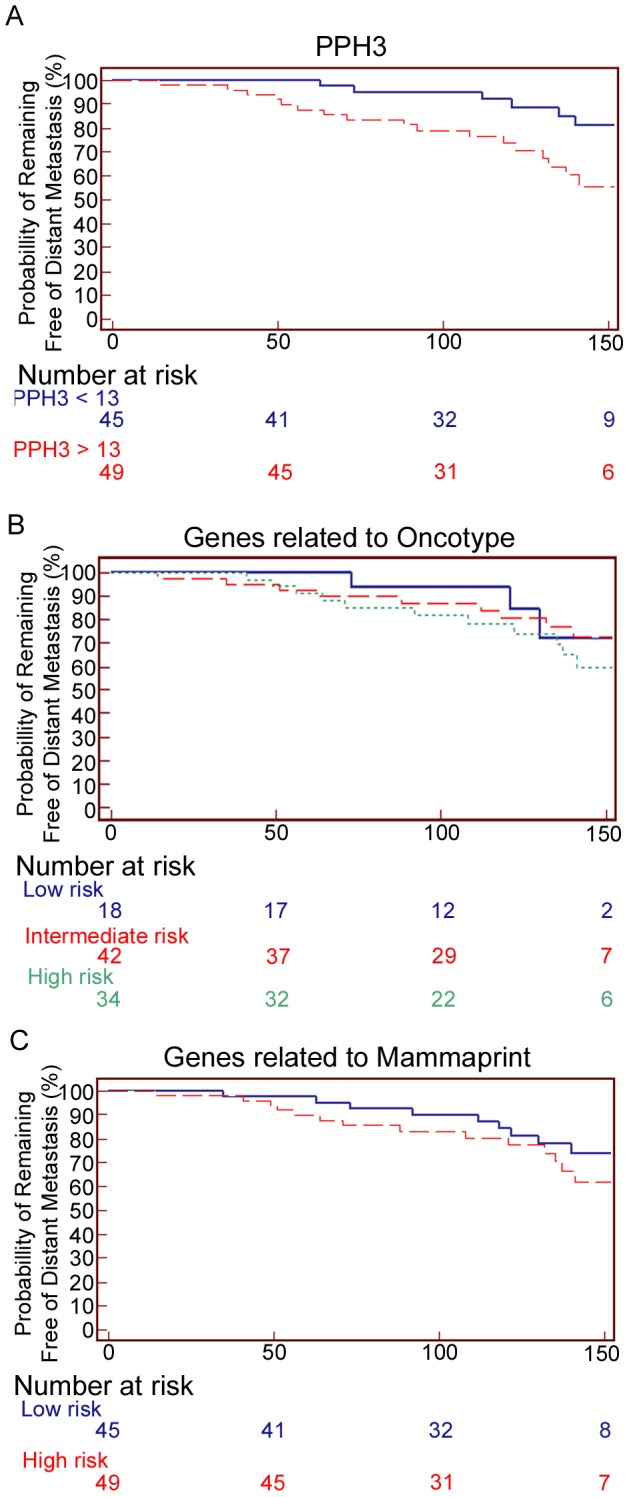
Long-term distant-metastasis-free survival curves according to PPH3 expression (A), classification by Oncotype DX RS (B), and classification by genes related to MammaPrint (C).

Kaplan-Meier survival analysis revealed that a high expression of PPH3 significantly identified patients who develop distant metastasis (Figure 1). Patients with high and low expressions of PPH3 had 14-year survival rate of a 65% and 87%, respectively (*p* = 0.014, HR = 3.1, 95% CI = 1.2–7.8). To test the prognostic value of the MammaPrint genes, the patients were classified into groups with low and high risks of DMFS based on the expression of these genes using hierarchical cluster analysis (Figure S1). Kaplan-Meier survival analysis of MammaPrint grouping resulted in a DMFS rates of 80% and 71% for low- and high-risk patients, respectively (*p* = 0.287, HR = 1.6, 95% CI = 0.7–3.6) with a 14-year follow-up. Furthermore, to validate the prognostic value of the 16 target genes related to Oncotype DX classification, the patients were divided into low-, intermediate- and high-risk groups according to their expression of these genes and based on ROC-curve analysis of RS (Figure S2); this categorization resulted in survival rates of 83% (*n* = 18), 79% (*n* = 42) and 68% (*n* = 34), respectively (*p* = 0.522, HR = 1.2 and 1.8, 95% CI = 0.3–4.4 and 0.5–6.5) with a 14-year follow-up. Originally, the Oncotype DX assay was designed for use in ERα-positive patients. Therefore Kaplan-Meier survival analysis was also performed in the subgroup of ERα-positive patients (*n* = 77), which yielded survival rates of 83%, 81% and 61% in the low-, intermediate- and high-risk groups, respectively (*p* = 0.293).

**Figure 2:**
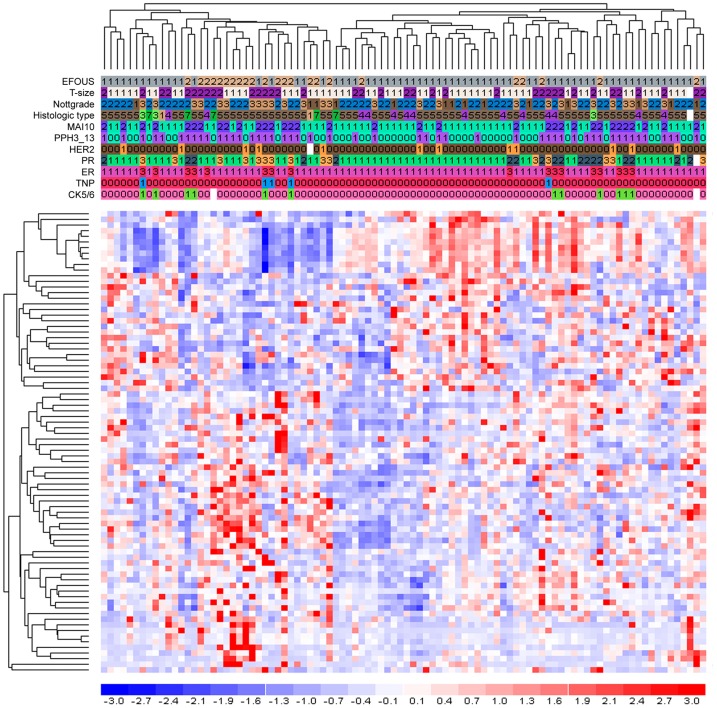
Supervised hierarchical clustering for DMFS. Each row represents an mRNA and each column represents a patient sample. The mRNA clustering tree is shown on the left, and the sample clustering tree appears at the top. The colour scale shown at the bottom illustrates the relative expression level of an mRNA across all samples: red colour represents an expression level above mean, blue colour represents expression lower than the mean. Gray colour means that the specific mRNA has not been successfully detected with microarray. Numbers for clinicopathological features indicate the following: EOFUS (1 =  no distant metastasis, 2 =  distant metastasis), Tsize (Tumour size: 1≤2 cm, 2 >2 cm) and Nottgrade (Nottingham grade: 1 =  grade 1, 2 =  grade 2, 3 =  grade 3), Histologic type (1 =  Tubular, 2 =  Colloid, 3 =  Medullary, 4 =  Lobular, 5 =  Ductal, 6 =  mix Ductal/Lobular, 7 =  Others), MAI10 (1<10, 2≥10), PPH3_13 (0<13, 1≥13), HER2 (0 = 0 or 1+, 1 = 2+ or 3+), PR (1<1%, 2 = 1–9% positive tumour cells, 3≥10% positive tumour cells), ERα (1<1% positive tumour cells, 3≥1% positive tumour cells), TNP (0 =  positive for either ERα/PR/Her2, 1 =  negative for ERα and PR and HER2), and CK5/6 (0 =  no staining, 1 =  any percentage of positive tumour cells).

ANOVA-analysis of all genes included in the Illumina WG6 array versus presence of distant metastasis or not (survival time was not taken into account) revealed that 82 genes were significantly (*p* = 0.01) associated with the presence or not of distant metastases (Figure 2); these genes are listed in Table S3 in File S1. The cross-validation (by leaving one sample out and reselecting genes) of overall classification accuracy was found to be 67% for these 82 genes. The same analysis showed that the sensitivity and specificity were 70% and 57%, respectively. The known biological processes associated with these 82 genes are also listed in Table 2. Genes related to pregnancy, fatty-acid metabolic processes and the regulation of growth are included in this signature. Furthermore, three of the genes were also found among the genes from MammaPrint (those encoding endothelial cell-specific molecule 1, origin recognition complex subunit 6 and Ras-related protein Rab-6B: ESM1, ORC6L and RAB6B, respectively).

**Table 2:** List of gene-ontology terms related to 82-gene signatures and presence or not of distant metastases.

Term	Genes involved	P-Value
Female pregnancy	PGF; placental growth factor;PSG3; pregnancy specific beta-1-glycoprotein 3; PSG4; pregnancy specific beta-1-glycoprotein 7; pregnancy specific beta-1-glycoprotein 8; pregnancy specific beta-1-glycoprotein 4 RG Homo sapiens; PSG9 pregnancy specific beta-1-glycoprotein 9; TRO; trophinin	7.1E-4
Fatty acid metabolic process	ACSL5; acyl-CoA synthetase long-chain family member 5; ADIPOR1; adiponectin receptor 1; C9ORF3; chromosome 9 open reading frame 3; PLA2G10; phospholipase A2, group X	3.7E-2
Regulation of growth	NELL2; NEL-like 2 (chicken); ADIPOR1; adiponectin receptor 1; ESM1; endothelial cell-specific molecule 1; FOXS1; forkhead box S1 RG Homo sapiens; TRO; trophinin	3.7E-2
rRNA processing	EXOSC7; exosome component 7; RPL11 ribosomal protein L11; RPS7; ribosomal protein S7; ribosomal protein S7 pseudogene 11; ribosomal protein S7 pseudogene 4; ribosomal protein S7 pseudogene 10	4.5E-2
rRNA metabolic process	EXOSC7; exosome component 7 RG; RPL11; ribosomal protein L11; RPS7; ribosomal protein S7; ribosomal protein S7 pseudogene 11; ribosomal protein S7 pseudogene 4; ribosomal protein S7 pseudogene 10	4.9E-2
Ribosome biogenesis	EXOSC7; exosome component 7; RPL11; ribosomal protein L11; RPS7; ribosomal protein S7; ribosomal protein S7 pseudogene 11; ribosomal protein S7 pseudogene 4; ribosomal protein S7 pseudogene 10	7.5E-2
Defence response	F12; coagulation factor XII (Hageman factor); CCR6; cyclin L2; chemokine (C-C motif) receptor 6; LILRB3; leukocyte immunoglobulin-like receptor, subfamily B (with TM and ITIM domains), member 3; PSG3; pregnancy specific beta-1-glycoprotein 3; PSG4; pregnancy specific beta-1-glycoprotein 7; pregnancy specific beta-1-glycoprotein 8; pregnancy specific beta-1-glycoprotein 4; PSG9; pregnancy specific beta-1-glycoprotein 9	7.6E-2
Cellular response to hormone stimulus	ADIPOR1 adiponectin receptor 1; GCGR; glucagon receptor; PGF; placental growth factor	8.7E-2

In the same way, investigation of which genes were significantly associated with the presence or not of distant metastases in patients with high proliferation (PPH3≥13) revealed that genes involved in Notch signalling and cell adhesion were expressed at higher levels in patients with distant metastases (Table 3). Seventeen genes from this gene list were also included in the 82-gene signature list, while none were found among the MammaPrint or Oncotype DX genes. No significant pathways were identified in the DMFS-related genes in the 45 patients with low proliferation, among whom only 6 patients developed distant metastasis.

**Table 3:** List of gene-ontology terms related high expression of PPH3 and presence or not of distant metastases.

Term	Genes involved	P-Value
Notch signalling pathway	NOTCH2NL; Notch homolog 2 N-terminal like; NOTCH4; Notch homolog 4; HEYL; hairy/enhancer-of-split related with YRPW motif-like	9.8E-3
Regulation of muscle contraction	ARG2; arginase, type II RG Homo sapiens; MYL5; myosin, light chain 5, regulatory; TNNT1; troponin T type 1 (skeletal, slow)	1.8E-2
Cell morphogenesis involved in differentiation	NOTCH4; Notch homolog 4; NDN; necdin homolog; NTN1; netrin 1; PLA2G10; phospholipase A2, group X	3.3E-2
Negative regulation of muscle contraction	ARG2; arginase, type I; TNNT1; troponin T type 1 (skeletal, slow)	3.4E-2
Cell-cell adhesion	CD93; CD93 molecule; FAT3; FAT tumour suppressor homolog 3; ESAM; endothelial cell adhesion molecule; PCDHB12; protocadherin beta 12	4.5E-2
Cell adhesion	CD93; CD93 molecule; FAT3; FAT tumour suppressor homolog 3; NELL2; NEL-like 2; COL5A3; collagen, type V, alpha 3 ESAM; endothelial cell adhesion molecule; PCDHB12 protocadherin beta 12	4.9E-2
Biological adhesion	CD93; CD93 molecule; FAT3; FAT tumour suppressor homolog 3; NELL2; NEL-like 2; COL5A3; collagen, type V, alpha 3; ESAM; endothelial cell adhesion molecule; PCDHB12; protocadherin beta 12	4.9E-2
Homophilic cell adhesion	FAT3; FAT tumour suppressor homolog 3; ESAM endothelial cell adhesion molecule; PCDHB12 protocadherin beta 12	5.5E-2
Regulation of system process	S100P S100 calcium binding protein P; ARG2 arginase, type II; MYL5 myosin, light chain 5, regulatory; TNNT1 troponin T type 1 (skeletal, slow)	5.9E-2
Regulation of growth	NELL2; NEL-like 2; FOXS1; forkhead box S1; NDN; necdin homolog; NTN1; netrin 1	7.5E-2
Cell morphogenesis	NOTCH4; Notch homolog 4; NDN; necdin homolog; NTN1; netrin; PLA2G10; phospholipase A2, group X	8.2E-2
Cell rojection organization	FGD5; FYVE, RhoGEF and PH domain containing 5; NDN; necdin homolog; NTN1; netrin 1; PLA2G10; phospholipase A2, group X	8.9E-2

Both forward and backward multivariate analysis of all significant features from Table 1 showed that PPH3 was the strongest independent prognostic factor.

## Discussion

This pilot study compared gene-expression data with the proliferation markers MAI, Ki67 and PPH3 status. Proliferation was the strongest independent prognostic factor in this cohort of breast cancer patients. Furthermore, in the group of patients with highly proliferative tumours, several genes related to the Notch signalling pathway appeared to add prognostic value.

Investigating expression data of groups of genes rather than single genes is believed to be a more reliable indicator of clinical response. Retrospective studies of the Oncotype DX assay have shown that it predicts recurrence better than classical clinicopathological variables [7], [29]. The assay is therefore widely requested by physicians, especially in North America, and studies have shown that the application of this assay changes patient management in 30% of cases [30]. The assay is currently only available in centralized laboratories, and is much more expensive than IHC. Evaluation of the Oncotype DX assay with a combined ER, PR, HER2 and Ki67 IHC score revealed a correlation coefficient of 0.7 [31]. Direct comparison of mRNA level [measured with quantitative polymerase chain reaction (qPCR)] and protein levels of ER, PR and HER2 (measured with IHC) revealed a high concordance value (79–94%) [32], while several other studies have found that a proliferation score based on PPH3 or Ki67 counts was significantly correlated with RS [33]–[35]. On the other hand, some have suggested that none of the standard clinicopathological features can accurately predict the RS [36]. Nevertheless, a meta-analysis of 647 ERα-positive patients with information about clinicopathological features and the RS demonstrated that the combined use of all of this information was more powerful than RS alone [37]. This combination also resulted in fewer patients being categorized in the intermediate-risk group, whereas up to 66% were classified as being at intermediate risk by RS alone. The present analysis of Oncotype-DX-related genes showed that although the survival rate appeared to be lower and the number of patients who suffered a recurrence appeared to be higher for the high-risk group than for the low-risk group, the differences were not statistically significant.

There are some technical differences between the present study and the original Oncotype DX assay, which was designed to detect the gene expression of mRNA isolated from formalin-fixed, paraffin-embedded (FFPE) material by means of qPCR. In the current study mRNA was isolated from fresh-frozen tumours and the gene expression was analysed using microarray technology. These technical differences do not seem to impair comparisons between the survival rates, since a paired comparison of gene expression between FFPE and fresh-frozen tissue yielded a strong correlation [38]. Furthermore, a strong correlation was also observed between microarray and qPCR findings [39], indicating that the present results are comparable to those of other studies that have employed Oncotype DX.

The 70-gene MammaPrint signature can divide patients into two separate groups: those with low and high risks of distant metastasis. The first study of this signature showed very good separation of the patients into these two groups, with HR and 95% CI values for the time to distant metastasis of 4.6 and 2.3–9.2, respectively, at a 10-year follow-up [8]. A validation study of the 70-gene profile signature showed that the assay is strongly prognostic, although the magnitude of the effect was much less than previously reported, with HR and 95% CI values for the time to distant metastases now being 2.32 and 1.35–4.00, respectively [40]. By comparison, a large multicentre prospective study exploring the prognostic value of the MAI found HR and 95% CI values for remaining free of distant metastasis of 3.12 and 2.17–4.50, respectively, thereby exceeding the prognostic value of the 70-gene signature [41]. Analyses of the prognostic information that lies in the 70-gene signature and other multigene signatures have shown that a large portion of the prognostic information lies in proliferation-related genes [42]. In fact, reanalyses of these signatures showed that the signature with proliferation-related genes had greater prognostic value than the original signature [43]–[45], and that the proliferation signature was correlated with the MAI (correlation coefficient, 0.968) [44]. One study showed that the non-proliferative genes had no prognostic power [43]. Reanalysis of the data from van't Veer et al. [5] showed that the molecular signature was strongly dependent on the selection of patients and that the set of genes was therefore not unique [46]. The present results with MammaPrint-related genes suggest that the proportion of patients with recurrence would be higher in the high-risk group than in the low-risk group. However, none of the analyses produced statistically significant results. The original MammaPrint assay was performed at a centralized laboratory and with a different microarray platform. Differences in the platforms used could influence the results; however, one study has shown that the Illumina and Affimetrix platforms are highly comparable [47]. Differences in the computation of the risk scores and definitions of thresholds by van't Veer et al. and in the present study could also be a possible reason for some of the discrepancies between the findings. Furthermore, not all of the genes were identified in the MammaPrint assay, which could have affected the statistical analysis.

Both the MammaPrint and Oncotype DX assays are based on RNA isolation from the tumour area, so that the harvested RNA is likely to be a mixture of both cancerous and normal tissue, and/or other non-cancerous cells. This may have affected the gene-expression profile and introduced significant bias into the prediction of the patient**s**' outcome [48]–[50]. Furthermore, the particular patient cohort included can affect the analysis and result in different gene signatures, as was the case in the current study; the gene signature of this study contained only three genes that were included in the MammaPrint signature, thereby confirming the observation that many different signatures can provide similar prognostic information, even in the same patient cohort [51].

Fresh-frozen tumour material for research is generally harvested from palpable and often larger tumours. Since tumour size is known to be related to prognosis, this may explain why the present cohort included a higher percentage of patients who developed distant metastasis.

The proliferation marker PPH3 is known to be a strong prognostic marker for LN-negative breast cancer; 35% of 49 patients with a high PPH3 suffered a recurrence. Although this means a high risk of developing distant metastasis, it also means that 65% did not develop distant metastasis. Therefore, it would be of great interest to investigate whether gene-expression analyses could add prognostic value to markers of proliferation. This could help to refine which patients really need chemotherapy, thereby decreasing both under- and overtreatment. In the present study, genes related to the Notch signalling pathway and Notch genes were expressed at significantly higher levels in patients with high proliferation rates who developed distant metastasis. The Notch family of cell-surface receptors is involved in proliferation, migration and invasion [52], [53]. Notch receptors have also been shown to regulate the self-renewal of mammary stem cells [54]. Furthermore, high Notch expression is observed in TNP, and is correlated with an overall poor outcome [55], [56]. In vivo knockdown experiments of Notch 1 and Notch 4 in tumour-bearing mice showed that the tumour size decreased and there was a reduction of breast cancer recurrence in these mice [53], [57]. Cell-line and in vivo experiments with ERα-positive breast cancer cells showed that tamoxifen treatment reactivated Notch signalling, which again induced proliferation and invasion. The same study showed that a combination of inhibition of Notch signalling and tamoxifen treatment inhibits tumour growth [52]. This observation has been confirmed by others [58].

In conclusion, the findings of this pilot study indicate that markers of proliferation status outperform MammaPrint- and Oncotype-DX-related genes as prognostic markers for LN-negative breast cancer. The inclusion of separate measurements of proliferation in future microarray studies might therefore be warranted. Furthermore, the obtained data support the previous finding that Notch could be a potential prognostic and predictive marker for the subgroup of highly proliferative LN-negative breast cancer patients.

## Supporting Information

Figure S1 Unsupervised cluster analysis with the genes related to MammaPrint assay. Colour codes: In the heat map (green colour indicate low expression of the mRNA and red indicates high expression), cluster prognosis (red  =  high risk of distant-metastasis, green  =  low risk of distant-metastasis), Distant metastasis (red  =  developing distant metastasis, green  =  no distant-metastasis), and correlation to average gene expression profile (AGPP) for no distant metastasis.
(TIF)

Figure S2 ROC-curve analysis for Oncotype DX RS and the optimal cut-offs.
(TIF)

File S1 Table S1, List and description of genes related to Oncotype DX. Table S2, List and description of genes related to MammaPrint assay. Table S3, List of 82-genes significant associated to DMFS.
(DOC)
