# Supplementary figures and images for: Prognostic Value of Gene Signatures and Proliferation in Lymph-Node-Negative Breast Cancer
Source: PLoS One. 2014 Mar 5;9(3):e90642. doi: 10.1371/journal.pone.0090642 (PMC3944091; doi:10.1371/journal.pone.0090642)

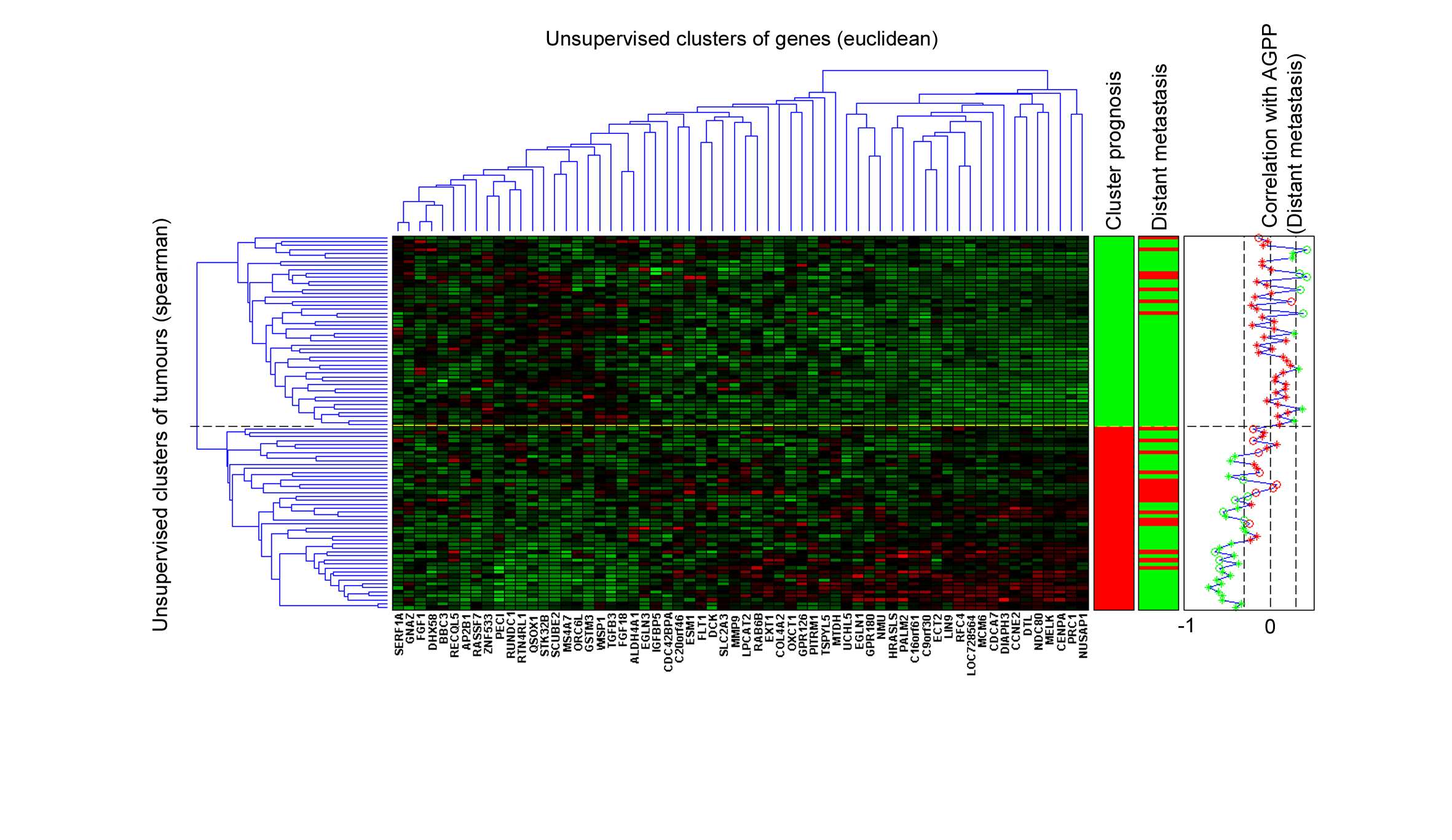

Supplement: Figure S1 — Unsupervised cluster analysis with the genes related to MammaPrint assay. Colour codes: In the heat map (green colour indicate low expression of the mRNA and red indicates high expression), cluster prognosis (red = high risk of distant-metastasis, green = low risk of distant-metastasis), Distant metastasis (red = developing distant metastasis, green = no distant-metastasis), and correlation to average gene expression profile (AGPP) for no distant metastasis. (TIF) [file pone.0090642.s001.tif]

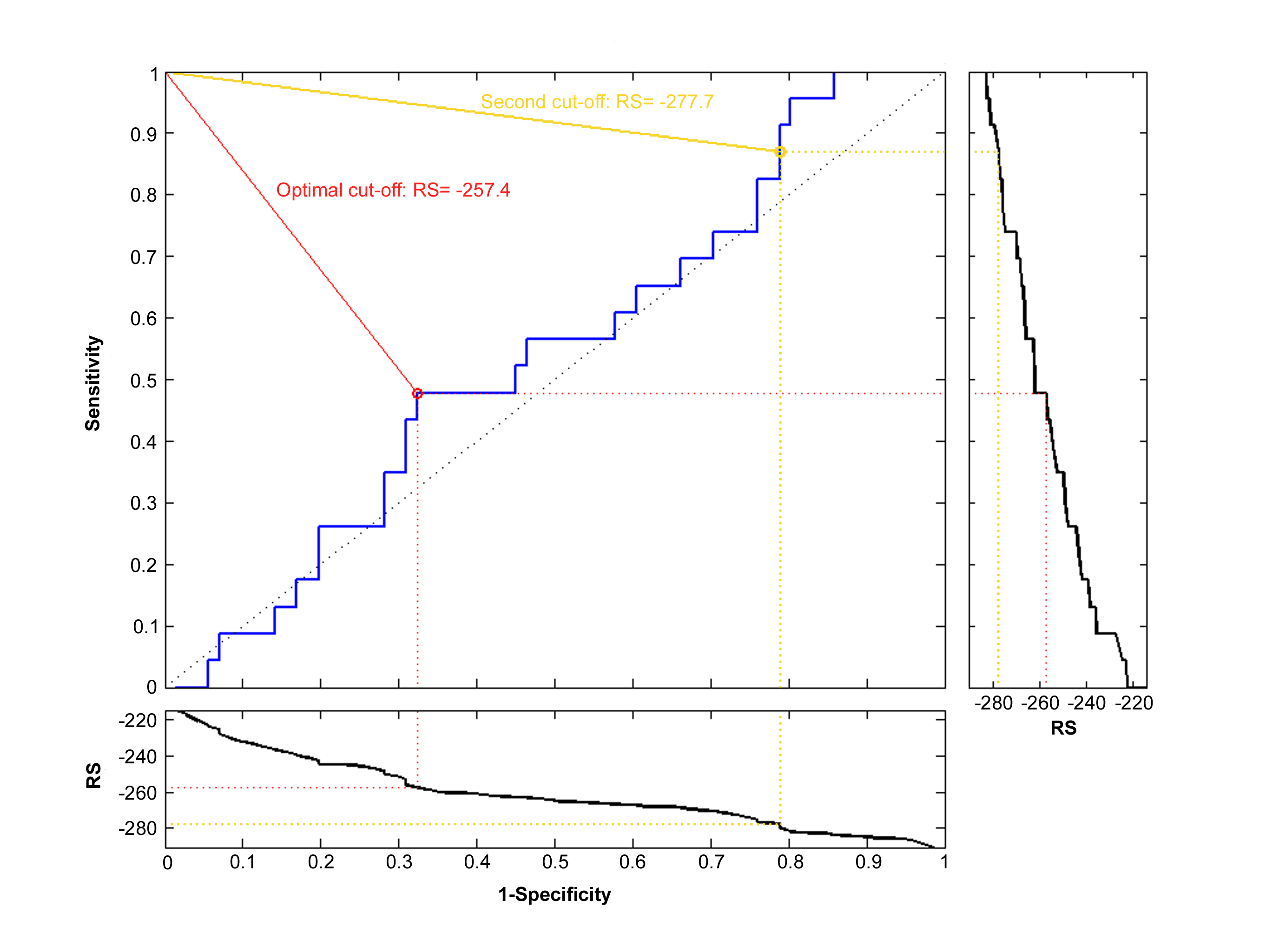

Supplement: Figure S2 — ROC-curve analysis for Oncotype DX RS and the optimal cut-offs. (TIF) [file pone.0090642.s002.tif]
