# Supplementary material for: Prognostic Value of Gene Signatures and Proliferation in Lymph-Node-Negative Breast Cancer
Source: PLoS One. 2014 Mar 5;9(3):e90642. doi: 10.1371/journal.pone.0090642 (PMC3944091; doi:10.1371/journal.pone.0090642)
Supplement: File S1 — Table S1, List and description of genes related to Oncotype DX. Table S2, List and description of genes related to MammaPrint assay. Table S3, List of 82-genes significant associated to DMFS. (DOC) [file pone.0090642.s003.doc]

**Table S1.** List and description of genes related to Oncotype DX.

| **Group** | **Oncotype Gene** | **Illumina gene name** | **Gene description** |
| --- | --- | --- | --- |
| Reference | ACTB (beta-actin) | ACTB | Homo sapiens actin, beta (ACTB), mRNA. |
|  | BAG1 | BAG1 | Homo sapiens BCL2-associated athanogene (BAG1), mRNA. |
| Estrogen | BCL2 | BCL2 | Homo sapiens B-cell CLL/lymphoma 2 (BCL2), nuclear gene encoding mitochondrial protein, transcript variant alpha, mRNA. |
| Proliferation | CCNB1 (cyclin B1) | CCNB1 | Homo sapiens cyclin B1 (CCNB1), mRNA. |
|  | CD68 | CD68 | Homo sapiens CD68 molecule (CD68), transcript variant 1, mRNA. |
|  | CTSL2 (Cathepsin L2) | CTSL2 | Homo sapiens cathepsin L2 (CTSL2), mRNA. |
| Estrogen | ER | ESR1 | Homo sapiens estrogen receptor 1 (ESR1), mRNA. |
| Reference | GAPDH | GAPDH | Homo sapiens glyceraldehyde-3-phosphate dehydrogenase (GAPDH), mRNA. |
| HER2 | GRB7 | GRB7 | Homo sapiens growth factor receptor-bound protein 7 (GRB7), transcript variant 1, mRNA. |
|  | GSTM1 | GSTM1 | Homo sapiens glutathione S-transferase M1 (GSTM1), transcript variant 1, mRNA. |
| Reference | GUS | GUSB | Homo sapiens glucuronidase, beta (GUSB), mRNA. |
| HER2 | HER2 | ERBB2 | Homo sapiens v-erb-b2 erythroblastic leukemia viral oncogene homolog 2, neuro/glioblastoma derived oncogene homolog (avian) (ERBB2), transcript variant 2, mRNA. |
| Proliferation | Ki67 | MKI67 | Homo sapiens antigen identified by monoclonal antibody Ki-67 (MKI67), mRNA. |
| Invasion | MMP11 (Stromolysin 3) | MMP11 | Homo sapiens matrix metallopeptidase 11 (stromelysin 3) (MMP11), mRNA. |
| Proliferation | MYBL2 | MYBL2 | Homo sapiens v-myb myeloblastosis viral oncogene homolog (avian)-like 2 (MYBL2), mRNA. |
| Estrogen | PGR | PGR | Homo sapiens progesterone receptor (PGR), mRNA. |
| Reference | RPLP0 | RPLP0 | Homo sapiens ribosomal protein, large, P0 (RPLP0), transcript variant 1, mRNA. |
| Estrogen | SCUBE2 | SCUBE2 | Homo sapiens signal peptide, CUB domain, EGF-like 2 (SCUBE2), mRNA. |
| Proliferation | STK15 | AURKA | Homo sapiens aurora kinase A (AURKA), transcript variant 5, mRNA. |
| Proliferation | STK15 | ZNF43 | Homo sapiens zinc finger protein 43 (ZNF43), mRNA. |
| Proliferation | Survivin | BIRC5 | Homo sapiens baculoviral IAP repeat-containing 5 (BIRC5), transcript variant 3, mRNA. |
| Reference | TFRC | TFRC | Homo sapiens transferrin receptor (p90, CD71) (TFRC), mRNA. |

**Table S2.** List and description of genes related to MammaPrint assay.

| **MammaPrint genes*** | **Ilummina name** | **Gene description** |
| --- | --- | --- |
| AA555029_RC | unknown |  |
| ALDH4A1 | ALDH4A1 | Homo sapiens aldehyde dehydrogenase 4 family, member A1 (ALDH4A1), nuclear gene encoding mitochondrial protein, transcript variant P5CDhL, mRNA. |
| AP2B1 | AP2B1 | Homo sapiens adaptor-related protein complex 2, beta 1 subunit (AP2B1), transcript variant 2, mRNA. |
| AYTL2 | LPCAT2 | Homo sapiens lysophosphatidylcholine acyltransferase 2 (LPCAT2), mRNA. |
| BBC3 | BBC3 | Homo sapiens BCL2 binding component 3 (BBC3), mRNA. |
| C16orf61 | C16orf61 | Homo sapiens chromosome 16 open reading frame 61 (C16orf61), mRNA. |
| C20orf46 | C20orf46 | Homo sapiens chromosome 20 open reading frame 46 (C20orf46), mRNA. |
| C9orf30 | C9orf30 | Homo sapiens chromosome 9 open reading frame 30 (C9orf30), mRNA. |
| CCNE2 | CCNE2 | Homo sapiens cyclin E2 (CCNE2), transcript variant 2, mRNA. |
| CDC42BPA | CDC42BPA | Homo sapiens CDC42 binding protein kinase alpha (DMPK-like) (CDC42BPA), transcript variant B, mRNA. |
| CDCA7 | CDCA7 | Homo sapiens cell division cycle associated 7 (CDCA7), transcript variant 1, mRNA. |
| CENPA | CENPA | Homo sapiens centromere protein A (CENPA), transcript variant 2, mRNA. |
| COL4A2 | COL4A2 | Homo sapiens collagen, type IV, alpha 2 (COL4A2), mRNA. |
| DCK | DCK | Homo sapiens deoxycytidine kinase (DCK), mRNA. |
| DIAPH3 | DIAPH3 | Homo sapiens diaphanous homolog 3 (Drosophila) (DIAPH3), transcript variant 1, mRNA. |
| DTL | DTL | Homo sapiens denticleless homolog (Drosophila) (DTL), mRNA. |
| EBF4 | unknown |  |
| ECT2 | ECT2 | Homo sapiens epithelial cell transforming sequence 2 oncogene (ECT2), mRNA. |
| EGLN1 | EGLN1 | Homo sapiens egl nine homolog 1 (C. elegans) (EGLN1), mRNA. |
| EGLN3 | EGLN3 | Homo sapiens egl nine homolog 3 (C. elegans) (EGLN3), mRNA. |
| ESM1 | ESM1 | Homo sapiens endothelial cell-specific molecule 1 (ESM1), mRNA. |
| EXT1 | EXT1 | Homo sapiens exostoses (multiple) 1 (EXT1), mRNA. |
| FGF1 | FGF1 | Homo sapiens fibroblast growth factor 1 (acidic) (FGF1), transcript variant 1, mRNA. |
| FGF18 | FGF18 | Homo sapiens fibroblast growth factor 18 (FGF18), mRNA. |
| FLT1 | FLT1 | Homo sapiens fms-related tyrosine kinase 1 (vascular endothelial growth factor/vascular permeability factor receptor) (FLT1), mRNA. |
| GMPS | LOC728564 | PREDICTED: Homo sapiens similar to GMP synthase [glutamine-hydrolyzing] (Glutamine amidotransferase) (GMP synthetase) (LOC728564), mRNA. |
| GNAZ | GNAZ | Homo sapiens guanine nucleotide binding protein (G protein), alpha z polypeptide (GNAZ), mRNA. |
| GPR126 | GPR126 | Homo sapiens G protein-coupled receptor 126 (GPR126), transcript variant a2, mRNA. |
| GPR180 | GPR180 | Homo sapiens G protein-coupled receptor 180 (GPR180), mRNA. |
| GSTM3 | GSTM3 | Homo sapiens glutathione S-transferase M3 (brain) (GSTM3), mRNA. |
| JHDM1D | unknown |  |
| HRASLS | HRASLS | Homo sapiens HRAS-like suppressor (HRASLS), mRNA. |
| IGFBP5 | IGFBP5 | Homo sapiens insulin-like growth factor binding protein 5 (IGFBP5), mRNA. |
| KNTC2 | NDC80 | Homo sapiens NDC80 homolog, kinetochore complex component (S. cerevisiae) (NDC80), mRNA. |
| LGP2 | DHX58 | Homo sapiens DEXH (Asp-Glu-X-His) box polypeptide 58 (DHX58), mRNA. |
| LIN9 | LIN9 | Homo sapiens lin-9 homolog (C. elegans) (LIN9), mRNA. |
| LOC100131053 | unknown |  |
| LOC100288906 | unknown |  |
| LOC730018 | unknown |  |
| MCM6 | MCM6 | Homo sapiens minichromosome maintenance complex component 6 (MCM6), mRNA. |
| MELK | MELK | Homo sapiens maternal embryonic leucine zipper kinase (MELK), mRNA. |
| MMP9 | MMP9 | Homo sapiens matrix metallopeptidase 9 (gelatinase B, 92kDa gelatinase, 92kDa type IV collagenase) (MMP9), mRNA. |
| MS4 A7 | MS4A7 | Homo sapiens membrane-spanning 4-domains, subfamily A, member 7 (MS4A7), transcript variant 2, mRNA. |
| MTDH | MTDH | Homo sapiens metadherin (MTDH), mRNA. |
| NMU | NMU | Homo sapiens neuromedin U (NMU), mRNA. |
| NUSAP1 | NUSAP1 | Homo sapiens nucleolar and spindle associated protein 1 (NUSAP1), transcript variant 2, mRNA. |
| ORC6L | ORC6L | Homo sapiens origin recognition complex, subunit 6 like (yeast) (ORC6L), mRNA. |
| OXCT1 | OXCT1 | Homo sapiens 3-oxoacid CoA transferase 1 (OXCT1), nuclear gene encoding mitochondrial protein, mRNA. |
| PALM2 | PALM2 | Homo sapiens paralemmin 2 (PALM2), transcript variant 2, mRNA. |
| PECI | PECI | Homo sapiens peroxisomal D3,D2-enoyl-CoA isomerase (PECI), transcript variant 1, mRNA. |
| PITRM1 | PITRM1 | Homo sapiens pitrilysin metallopeptidase 1 (PITRM1), mRNA. |
| PRC1 | PRC1 | Homo sapiens protein regulator of cytokinesis 1 (PRC1), transcript variant 2, mRNA. |
| QSCN6L1 | QSOX1 | Homo sapiens quiescin Q6 sulfhydryl oxidase 1 (QSOX1), transcript variant 1, mRNA. |
| RAB6B | RAB6B | Homo sapiens RAB6B, member RAS oncogene family (RAB6B), mRNA. |
| RASSF7 | RASSF7 | Homo sapiens Ras association (RalGDS/AF-6) domain family (N-terminal) member 7 (RASSF7), mRNA. |
| RECQL5 | RECQL5 | Homo sapiens RecQ protein-like 5 (RECQL5), transcript variant 1, mRNA. |
| RFC4 | RFC4 | Homo sapiens replication factor C (activator 1) 4, 37kDa (RFC4), transcript variant 2, mRNA. |
| RTN4RL1 | RTN4RL1 | Homo sapiens reticulon 4 receptor-like 1 (RTN4RL1), mRNA. |
| RUNDC1 | RUNDC1 | Homo sapiens RUN domain containing 1 (RUNDC1), mRNA. |
| SCUBE2 | SCUBE2 | Homo sapiens signal peptide, CUB domain, EGF-like 2 (SCUBE2), mRNA. |
| SERF1A | SERF1A | Homo sapiens small EDRK-rich factor 1A (telomeric) (SERF1A), mRNA. |
| SLC2A3 | SLC2A3 | Homo sapiens solute carrier family 2 (facilitated glucose transporter), member 3 (SLC2A3), mRNA. |
| STK32B | STK32B | Homo sapiens serine/threonine kinase 32B (STK32B), mRNA. |
| TGFB3 | TGFB3 | Homo sapiens transforming growth factor, beta 3 (TGFB3), mRNA. |
| TSPYL5 | TSPYL5 | Homo sapiens TSPY-like 5 (TSPYL5), mRNA. |
| UCHL5 | UCHL5 | Homo sapiens ubiquitin carboxyl-terminal hydrolase L5 (UCHL5), mRNA. |
| WISP1 | WISP1 | Homo sapiens WNT1 inducible signaling pathway protein 1 (WISP1), transcript variant 1, mRNA. |
| ZNF533 | ZNF533 | Homo sapiens zinc finger protein 533 (ZNF533), mRNA. |

*Gene list from [24].

**Table S3.** List of 82-genes significant associated to DMFS

| **Gene symbole** | **Description** |
| --- | --- |
| ACSL5 | Homo sapiens acyl-CoA synthetase long-chain family member 5 (ACSL5), transcript variant 1, mRNA. |
| ADHFE1 | Homo sapiens alcohol dehydrogenase, iron containing, 1 (ADHFE1), nuclear gene encoding mitochondrial protein, mRNA. |
| ADIPOR1 | Homo sapiens adiponectin receptor 1 (ADIPOR1), mRNA. |
| ANGPT2 | Homo sapiens angiopoietin 2 (ANGPT2), mRNA. |
| ARHGEF6 | Homo sapiens Rac/Cdc42 guanine nucleotide exchange factor (GEF) 6 (ARHGEF6), mRNA. |
| BLVRA | Homo sapiens biliverdin reductase A (BLVRA), mRNA. |
| C17ORF82 | Homo sapiens chromosome 17 open reading frame 82 (C17orf82), mRNA. |
| C19ORF31 | Homo sapiens chromosome 19 open reading frame 31 (C19orf31), mRNA. |
| C1ORF109 | Homo sapiens chromosome 1 open reading frame 109 (C1orf109), mRNA. |
| C9ORF169 | Homo sapiens chromosome 9 open reading frame 169 (C9orf169), mRNA. |
| C9ORF3 | Homo sapiens chromosome 9 open reading frame 3 (C9orf3), mRNA. |
| CCR6 | Homo sapiens chemokine (C-C motif) receptor 6 (CCR6), transcript variant 2, mRNA. |
| CD93 | Homo sapiens CD93 molecule (CD93), mRNA. |
| CEACAM5 | Homo sapiens carcinoembryonic antigen-related cell adhesion molecule 5 (CEACAM5), mRNA. |
| CEACAM6 | Homo sapiens carcinoembryonic antigen-related cell adhesion molecule 6 (non-specific cross reacting antigen) (CEACAM6), mRNA. |
| CLUAP1 | Homo sapiens clusterin associated protein 1 (CLUAP1), transcript variant 2, mRNA. |
| CNTD2 | Homo sapiens cyclin N-terminal domain containing 2 (CNTD2), mRNA. |
| COL4A1 | Homo sapiens collagen, type IV, alpha 1 (COL4A1), mRNA. |
| COMTD1 | Homo sapiens catechol-O-methyltransferase domain containing 1 (COMTD1), mRNA. |
| CXCR7 | Homo sapiens chemokine (C-X-C motif) receptor 7 (CXCR7), mRNA. |
| CYB561D2 | Homo sapiens cytochrome b-561 domain containing 2 (CYB561D2), mRNA. |
| EPB41L4B | Homo sapiens erythrocyte membrane protein band 4.1 like 4B (EPB41L4B), transcript variant 1, mRNA. |
| ESM1 | Homo sapiens endothelial cell-specific molecule 1 (ESM1), mRNA. |
| EXOSC7 | Homo sapiens exosome component 7 (EXOSC7), mRNA. |
| F12 | Homo sapiens coagulation factor XII (Hageman factor) (F12), mRNA. |
| FARSLB | Homo sapiens phenylalanine-tRNA synthetase-like, beta subunit (FARSLB), mRNA. |
| FLJ35801 | Homo sapiens hypothetical protein FLJ35801 (FLJ35801), mRNA. |
| FLJ41603 | Homo sapiens FLJ41603 protein (FLJ41603), mRNA. |
| FOXS1 | Homo sapiens forkhead box S1 (FOXS1), mRNA. |
| FXYD3 | Homo sapiens FXYD domain containing ion transport regulator 3 (FXYD3), transcript variant 1, mRNA. |
| GCGR | Homo sapiens glucagon receptor (GCGR), mRNA. |
| GPC6 | Homo sapiens glypican 6 (GPC6), mRNA. |
| GTF2F1 | Homo sapiens general transcription factor IIF, polypeptide 1, 74kDa (GTF2F1), mRNA. |
| H6PD | Homo sapiens hexose-6-phosphate dehydrogenase (glucose 1-dehydrogenase) (H6PD), mRNA. |
| HECTD2 | Homo sapiens HECT domain containing 2 (HECTD2), transcript variant 1, mRNA. |
| HEYL | Homo sapiens hairy/enhancer-of-split related with YRPW motif-like (HEYL), mRNA. |
| HS.192784 | Homo sapiens cDNA FLJ36653 fis, clone UTERU2001176 |
| HS.434957 | Homo sapiens, clone IMAGE:3618365, mRNA |
| HS.537292 | Homo sapiens mRNA; cDNA DKFZp686F16120 (from clone DKFZp686F16120) |
| IAH1 | Homo sapiens isoamyl acetate-hydrolyzing esterase 1 homolog (S. cerevisiae) (IAH1), mRNA. |
| ITGB4 | Homo sapiens integrin, beta 4 (ITGB4), transcript variant 2, mRNA. |
| ITIH5 | Homo sapiens inter-alpha (globulin) inhibitor H5 (ITIH5), transcript variant 1, mRNA. |
| KIAA0319 | Homo sapiens KIAA0319 (KIAA0319), transcript variant 1, mRNA. |
| KIAA1107 | PREDICTED: Homo sapiens KIAA1107 (KIAA1107), mRNA. |
| KIAA1199 | Homo sapiens KIAA1199 (KIAA1199), mRNA. |
| LILRB3 | Homo sapiens leukocyte immunoglobulin-like receptor, subfamily B (with TM and ITIM domains), member 3 (LILRB3), transcript variant 2, mRNA. |
| LOC400721 | PREDICTED: Homo sapiens similar to Zinc finger protein 418 (LOC400721), mRNA. |
| LOC645287 | PREDICTED: Homo sapiens similar to Group X secretory phospholipase A2 precursor (Phosphatidylcholine 2-acylhydrolase GX) (GX sPLA2) (sPLA2-X) (LOC645287), mRNA. |
| LOC649456 | PREDICTED: Homo sapiens similar to Kinase suppressor of ras-1 (Kinase suppressor of ras) (LOC649456), mRNA. |
| LRFN5 | Homo sapiens leucine rich repeat and fibronectin type III domain containing 5 (LRFN5), mRNA. |
| MC1R | Homo sapiens melanocortin 1 receptor (alpha melanocyte stimulating hormone receptor) (MC1R), mRNA. |
| MICAL1 | Homo sapiens microtubule associated monoxygenase, calponin and LIM domain containing 1 (MICAL1), mRNA. |
| MXD3 | Homo sapiens MAX dimerization protein 3 (MXD3), mRNA. |
| N4BP2L1 | Homo sapiens NEDD4 binding protein 2-like 1 (N4BP2L1), transcript variant 2, mRNA. |
| NELL2 | Homo sapiens NEL-like 2 (chicken) (NELL2), mRNA. |
| NEURL4 | Homo sapiens neuralized homolog 4 (Drosophila) (NEURL4), transcript variant 1, mRNA. |
| ORC6L | Homo sapiens origin recognition complex, subunit 6 like (yeast) (ORC6L), mRNA. |
| PATE2 | Homo sapiens prostate and testis expressed 2 (PATE2), mRNA. |
| PCDH17 | Homo sapiens protocadherin 17 (PCDH17), mRNA. |
| PFAS | Homo sapiens phosphoribosylformylglycinamidine synthase (FGAR amidotransferase) (PFAS), mRNA. |
| PGF | Homo sapiens placental growth factor (PGF), mRNA. |
| PLA2G10 | Homo sapiens phospholipase A2, group X (PLA2G10), mRNA. |
| PRND | Homo sapiens prion protein 2 (dublet) (PRND), mRNA. |
| PSG3 | Homo sapiens pregnancy specific beta-1-glycoprotein 3 (PSG3), mRNA. |
| PSG4 | Homo sapiens pregnancy specific beta-1-glycoprotein 4 (PSG4), transcript variant 1, mRNA. |
| PSG9 | Homo sapiens pregnancy specific beta-1-glycoprotein 9 (PSG9), mRNA. |
| PTRH2 | Homo sapiens peptidyl-tRNA hydrolase 2 (PTRH2), nuclear gene encoding mitochondrial protein, mRNA. |
| RAB5C | Homo sapiens RAB5C, member RAS oncogene family (RAB5C), transcript variant 1, mRNA. |
| RPL11 | Homo sapiens ribosomal protein L11 (RPL11), mRNA. |
| RPS7 | Homo sapiens ribosomal protein S7 (RPS7), mRNA. |
| S100P | Homo sapiens S100 calcium binding protein P (S100P), mRNA. |
| SALL3 | Homo sapiens sal-like 3 (Drosophila) (SALL3), mRNA. |
| SCGB1C1 | Homo sapiens secretoglobin, family 1C, member 1 (SCGB1C1), mRNA. |
| SGSM2 | Homo sapiens small G protein signaling modulator 2 (SGSM2), transcript variant 1, mRNA. |
| SLC6A1 | Homo sapiens solute carrier family 6 (neurotransmitter transporter, GABA), member 1 (SLC6A1), mRNA. |
| SSBP2 | Homo sapiens single-stranded DNA binding protein 2 (SSBP2), mRNA. |
| TRO | Homo sapiens trophinin (TRO), transcript variant 6, mRNA. |
| TSPAN6 | Homo sapiens tetraspanin 6 (TSPAN6), mRNA. |
| UBTD1 | Homo sapiens ubiquitin domain containing 1 (UBTD1), mRNA. |
| WDR57 | Homo sapiens WD repeat domain 57 (U5 snRNP specific) (WDR57), mRNA. |
| ZBBX | Homo sapiens zinc finger, B-box domain containing (ZBBX), mRNA. |
| ZMYND12 | Homo sapiens zinc finger, MYND-type containing 12 (ZMYND12), mRNA. |
